# Chitosan Membrane Modified With a New Zinc(II)-Vanillin Complex Improves Skin Wound Healing in Diabetic Rats

**DOI:** 10.3389/fphar.2018.01511

**Published:** 2019-01-08

**Authors:** Emanuella de Aragão Tavares, Wendy Marina Toscano Queiroz de Medeiros, Talita Pereira de Assis Pontes, Maisie Mitchele Barbosa, Aurigena Antunes de Araújo, Raimundo Fernandes de Araújo, Jozi Godoy Figueiredo, Renata Carvalho Leitão, Conceição da Silva Martins, Francisco Ordelei Nascimento da Silva, Ana Cristina Facundo de Brito Pontes, Daniel de Lima Pontes, Caroline Addison Carvalho Xavier de Medeiros

**Affiliations:** ^1^Post Graduation Program in Biological Sciences, Federal University of Rio Grande doNorte, Natal, Brazil; ^2^Institute of Chemistry, Federal University of Rio Grande do Norte, Natal, Brazil; ^3^Post Graduation Program in Biotechnology RENORBIO, Federal University of Rio Grande do Norte, Natal, Brazil; ^4^Post Graduation Program Public Health/Post Graduation Program in Pharmaceutical Sciences, Department of Biophysics and Pharmacology, Federal University of Rio Grande do Norte, Natal, Brazil; ^5^Post Graduation Program in Functional and Structural Biology/Post Graduation Program Health Sciences, Department of Morphology, Federal University of Rio Grande do Norte, Natal, Brazil; ^6^Institute of Biotechnology, University of Caxias do Sul, Caxias do Sul, Brazil; ^7^Post Graduation Program of Morphological Science, Department of Morphology, Universidade Federal do Ceará, Fortaleza, Brazil; ^8^Post Graduation Program in Biological Sciences/Post Graduation Program in Biotechnology RENORBIO, Department of Biophysics and Pharmacology, Federal University of Rio Grande do Norte, Natal, Brazil

**Keywords:** diabetes, skin lesions, zinc, chitosan membrane, healing

## Abstract

The treatment of chronic wounds is considered a public health problem. When the condition affects at-risk groups such as those with diabetics, it becomes a great clinical challenge. In this work, we evaluated the healing effects of a new zinc complex, [Zn(phen)(van)_2_], identified as ZPV, which was synthesized, characterized and associated with chitosan (CS) membranes and tested on cutaneous wounds of diabetic rats. Chitosan membranes were modified by Schiff base reaction with the complex under two experimental conditions (14 and 21 days), resulting in membranes with concentrations of complex equal to 0.736 μmol cm^-2^ (CS-ZPV1) and 1.22 μmol cm^-2^ (CS-ZPV2). Release assays in aqueous medium indicated that the membranes release the complex gradually when exposed to an aqueous medium. Diabetes was inducted in *Wistar* rats using 40 mg/kg (i.v.) streptozotocin. On the 7th day after diabetic induction, a circular excision on the skin (1.0 cm) was performed with a punch. The lesions were treated with the pure chitosan membrane and the membrane associated with the zinc-vanillin complex in two different doses. Skin samples were subjected to macroscopic and histopathological analyses, cytokine (TNF-α, IL-1β, and IL-10) quantification and reverse transcriptase polymerase chain reaction (TGF-β and VEGF) assays. The analyses showed a decrease in wound size, reepithelialization, angiogenic stimulus, collagen deposition, and reduced levels of TNF-α and IL-1β as well as increased IL-10 and gene expression of TGF-β and VEGF. The evaluated parameters suggest that CS-ZPV in the two concentrations tested may be effective in the treatment of chronic wounds.

## Introduction

Diabetes mellitus is a serious disease characterized by high levels of glucose plasma, and it represents a serious public health problem with negative impacts on the quality of life of patients. Moreover, the global prevalence for diabetes is estimated to be increasing and expected to reach 642 million adults by 2040 (Wu et al., 2014). Hyperglycaemia has toxic effects on many cells in the body, contributing to the appearance of acute and chronic complications associated with the disease.

Diabetic foot ulcers are a complication with high incidence usually involved with hospitalizations, and they can often lead to lower-extremity amputations. Diabetic patients present late or poor wound healing, and elevated glucose may induce oxidative stress and activate inflammatory response capacity for the development of diabetic neuropathy. Diabetics with a diabetic neuropathy are more likely to develop serious wounds with complications (Falanga, 2005).

Zinc(II) is an essential micronutrient that plays an important role in the wound-healing process. This metal is a cofactor for many metalloenzymes required for cell membrane repair, cell proliferation, growth and immune system function (Lin et al., 2017). Although different coordination compounds having zinc(II) as a metal center are biologically active systems, with potential application as metallodrugs, the development of zinc complexes with healing properties has still been scarcely explored.

Among other key aspects to be considered in a metal complex, the ligand is also a critical part, and it might establish the biological function of the compound. Cellular uptake, organelle distribution, appropriated structural organization for biomolecule interactions and overall pharmacological activity can also be adjusted upon the selection of suitable ligands (Kaim and Schwederski, 2010; Romero-Canelon and Sadler, 2013; Romo et al., 2016).

Vanillin (vanH), a natural phenolic compound of vanilla orchids, is one of the most widely used flavoring agents in food, beverage and cosmetics (Wu et al., 2009). In addition to its industrial and economic value, vanillin has also been recognized as an important bioactive compound presenting a range of relevant biochemical and pharmacological activities, such as antioxidant, antimicrobial, antifungal and antitumor properties (De Flora et al., 1994; Shaughnessy et al., 2001; Tai et al., 2011).

There is a growing interest in the development of selective drug delivery systems (Wang and Wang, 2009). In this context, bioabsorbable polymers, such as chitosan, have received special attention in the biomedical area. Chitosan materials can be prepared as fibers, membranes, hydrogels, films, microspheres or capsules (Zou et al., 2015). The most relevant properties of chitosan are related to its biodegradability, biocompatibility, non-toxicity and commercial availability, in addition to presenting antifungal and antibacterial activity, which makes this biopolymer the target of studies that involve the regeneration of living tissues, wound healing and administration of drugs.

Thus, this work has as objectives the development, characterization and evaluation of a new complex of zinc(II), [Zn(phen)(van)_2_], administered through modification of the chitosan membrane in the experimental model of wounds in diabetic rats.

## Materials and Methods

### Material

The reagents anhydrous ZnCl_2_, 1,10-phenanthroline, vanillin, D-glucosamine hydrochloride and streptozotocin were purchased from Sigma-Aldrich. Chitosan was acquired from Polymar (Ceará, Brazil) and purified as described in the literature (Campana F^o^ and Signini, 2001). The precursor complex [Zn(phen)Cl_2_] was prepared according to a previously described procedure (Awad et al., 2010).

### Preparation of Chitosan Membranes

A solution of purified chitosan (1.0 g) solubilized in 50 mL of acetic acid (1%, v/v) was stirred for 24 h. After this period, 12.5 mL of this solution was added to 50 mL petri plates and let to stand at 25°C. The membrane originated after the complete evaporation of the solvent was washed with sodium hydroxide solution (1% w/v) and distillated water and dried at room temperature.

### Synthesis of the Complex [Zn(phen)(van)_2_] (ZPV)

The compound [Zn(phen)(van)_2_] was synthesized following procedure previously reported by our group for similar complex with copper(II) (de Medeiros et al., 2018). [Zn(phen)Cl_2_] (200.0 mg, 0.63 mmol) and vanillin (192.2 mg, 1.26 mmol) were dissolved in distilled water. The pH of the solution was adjusted to 8.0 by the addition of sodium hydroxide (2.0 mol L^-1^). This reaction mixture was stirred for 2 h at 60°C and a yellow solid formed, which was filtered and washed with ethanol. Yield: 272.7 mg (79%).

IR: υ_max_/cm^-1^; υ(C = O): 1660; υ(C = C): 1586, 1545; υ(C–CHO): 1261; υ(O–CH_3_): 1021; δ(C–H): 849, 727 (KBr disk). UV-Vis: λ(H_2_O)/nm (𝜀/L mol^-1^ cm^-1^): 345 (16000), 312 (sh) (17200), 271 (45900), 201 (55600). ^1^H NMR spectrum in DMSO-d6 (δ/ppm), 30°C: 9.57 (2H, s, CHO), 8.99–8.91 (2H, d, H-phen), 8.29 (2H, t, H-phen), 8.09 (2H, s, H-phen), 7.31–7.28 (2H, d, H-van), 7.24 (2H, s, H-van), 6.67 (2H, d, H-van), 3.71 (2H, s, OCH_3_).

### Synthesis of the Schiff Base 2-Deoxy-2-[(E)-(4-Hydroxy-3-Methoxybenzylidene)Amino] Hexopyranose (vglu)

The Schiff’s base was synthesized following procedure previously reported by similar compounds (Costa Pessoa et al., 2003). D-glucosamine⋅HCl (709.0 mg, 3.29 mmol) and sodium hydroxide (132.0 mg, 3.29 mmol) were dissolved in methanol and stirred for 20 min. The sodium chloride formed was filtered and vanillin (500.0 mg, 3.29 mmol) was solubilized in the solution and stirred for 2 h at 60°C. The obtained yellow solid was filtrated and washed with methanol. Yield: 886.5 mg (86%).

IR: υ_max_/cm^-1^; υ(C = N): 1635; υ(C = C): 1589, 1528; δ(C–H e O–H): 1298, 1248, 1207; υ(C-O_pyranose_): 1088; υ(O–CH_3_): 1035; (KBr disk). UV-Vis: λ(H_2_O)/nm (𝜀/L mol^-1^ cm^-1^): 208 (15 000), 227 (12 700), 271 (10 770), 303 (8 540), 393 (6 762). ^1^H NMR spectrum in DMSO (δ/ppm), 30°C: 9.57 (1H, s, H_imina_), 7.28–7.27 (1H, d, H-van), 7.20 (1H, s, H-van), 6.79–6.78 (1H, d, H-van), 3.99–3.84 (m, H-glu), 3.72 (1H, s, OCH_3_), 3.68–3.35 (m, H-glu).

### Preparation of Chitosan Membranes Modified With [Zn(phen)(van)_2_] (CS-ZPV1 and CS-ZPV2)

Square chitosan membranes (2.5 cm × 2.5 cm) were immersed in 5 mL of a [Zn(phen)(van)_2_] (0.01 mol L^-1^, 50:50 methanol/dimethyl sulfoxide (DMSO) solution. The reactional system was maintained at room temperature for 14 or 20 days to obtain membranes with different contents of the [Zn(phen)(van)_2_] complex, 0.403 mg cm^-2^ and 0.671 mg cm^-2^, respectively. The membranes were thoroughly washed with methanol / DMSO (50:50) solution and dried in a desiccator. The modified membranes obtained after 14 and 20 days of immersion in the solution of the complex were identified in this work as CS-ZPV1 and CS-ZPV2, respectively.

### Spectroscopic and Electrochemistry Characterization

The electronic spectra were obtained in water and PBS buffer (0.1 mol L^-1^, pH 7.4) at room temperature in a UV-Vis spectrometer, model 8453 from Agilent. The infrared spectra were performed from 4000 to 400 cm^-1^ in a Shimadzu FTIR-8400S. The powder metal complexes and ligands were dispersed in KBr pellets while the chitosan membranes were analyzed without previous treatment, allowing the infrared radiation to pass through the film.

An electrochemical analysis was performed on an Epsilon potentiostat (BASi – Bioanalytical Systems Inc.). Cyclic voltammetric experiments were performed in a three-electrode cell. The working electrode was a glassy carbon, a platinum single-wire electrode was used as the counter electrode and an Ag/AgCl electrode saturated with KCl (3.5 mol L^-1^) was used as the reference electrode. The cyclic voltammograms were recorded in 0.1 mol L^-1^ phosphate-buffered saline (PBS) solution, pH 7.4, at ν = 100 mV s^-1^. Oxygen was removed by purging the solutions with Argon. All measurements were performed at 25.0 ± 0.2°C.

The scanning electron microscope (SEM) images of chitosan membrane (CS), and modified chitosan with zinc complex (CS-ZPV) were obtained on an MEV – FEG (Zeiss) BRUKER e^-^flash. The working conditions were as follows: accelerating voltage 10.00 kV and WD (working distance) 3.9 and 4.0 mm. All samples were gold coated for 30 s at 30 mA prior to SEM examination.

The nuclear magnetic resonance (NMR) spectra were obtained on a spectrometer (Bruker Avance DRX, 300 MHz) at 303 K using dimethyl sulfoxide as the solvent.

### Determination of the [Zn(phen)(van)_2_] Content in the Modified Chitosan Membrane and Evaluation of Delivery

The [Zn(phen)(van)_2_] content in the modified chitosan membranes, CS-ZPV1 and CS-ZPV2, was determined by UV-Vis spectroscopy monitoring the release of the complex from the membrane. Square samples of the modified chitosan membranes with a total area of 0.08 cm^2^ were immersed in 3.2 mL of PBS (0.1 mol L^-1^, pH 7.4) and kept without stirring for 23 h. The release of the ZPV complex from the membranes was monitored by recording several spectra of the solution until stabilization of the absorbance at 228 nm, referring to a band present in the spectrum of the ZPV complex. The concentration of the complex was determined by a calibration curve prepared by solutions in 0.1 mol L^-1^ PBS, pH 7.4, of [Zn(phen)(van)_2_] from 6.0 × 10^-6^ mol L^-1^ to 1.6 × 10^-5^mol L^-1^.

The experiments were performed in triplicate, and the concentrations are given in relation to the membrane area evaluated as μmol cm^-2^ or mg cm^-2^. A qualitative monitoring of the ZPV release was also performed by cyclic voltammetry using PBS (0.1 mol L^-1^, pH 7.4) as the support electrolyte.

### Diabetes Induction and Cutaneous Wounds

Forty male *Wistar* rats weighing between 240–260 g were used. This project was approved by the Animal Experiment Ethics Committee of the Federal University of Rio Grande do Norte, protocol number (005006/2017). For the induction of diabetes, the animals were anesthetized by inhalation with isoflurane, and then 40 mg/kg streptozotocin by dissolved in 0.1 citrate buffer with a pH of 4.5 was administered by intravenous route (vein of the penis). Ninety-six hours after the administration of streptozotocin, the glycaemia was verified with the use of a glucose meter. Diabetics were diagnosed animals with blood glucose values higher than 300 mg/dL (Cunha et al., 2009).

Seven days after the diabetes induction procedure, the animals were anesthetized intraperitoneally with 80 mg/kg ketamine and 100 mg/kg and xylazine. After anesthesia, a trichotomy (hair removal) was performed in the dorsal region to facilitate good viewing of the operative field, as well as to facilitate the analysis of the healing process of the wounds. The area was again cleaned with 4% chlorhexidine, before a circular excision was made in the skin measuring 1.0 cm in diameter using a dermatological punch that removed a small piece of skin, exposing the *Panniculus carnosus*. The membrane was immediately applied over the wound. The rats were subjected to euthanasia with sodium thiopental (90 mg/kg, intraperitoneal via) on days 7 and 14 after the procedure (Martins et al., 2006).

### Experimental Groups

The rats were randomly divided into five groups (*n* = 5 per group): normal (without diabetes, with natural healing); diabetics (with diabetes, without membrane treatment), chitosan group (CS) (diabetic rats treated with pure chitosan membrane) and groups CS-ZPV (rats diabetics treated with CS-ZPV1 and CS-ZPV2 with concentrations of [Zn(phen)(van)_2_] of 0.403 mg cm^-2^ and 0.460 mg cm^-2^, respectively).

### Macroscopic and Histopathological Analysis

After euthanasia, at 7 or 14 days, a macroscopic analysis of the lesions on the dorsum of the rats was performed. The percentage area of the wound on the postoperative days was calculated by comparing the changes in wound size in relation to the first day of the procedure. The monitoring of cicatricial evolution was used in *ImageJ software* for measurement (de Avila Santana et al., 2013). After the macroscopic analysis, wounds were excised with 2 mm margin beyond the wound edge for histopathological evaluation, and a representative sample was used to illustrate the wound healing observed in the present study.

For histopathological analysis, tissue samples were fixed with 10% neutral buffered formalin, dehydrated with alcohol at different times and concentrations, embedded in paraffin and cutted at 5 μmin a microtome for staining with routine hematoxylin-eosin (HE) (Abramov et al., 2007), Mallory’s trichrome (Cheng et al., 2008) or picrosirius red (ScyTek^®^, Logan, UT, United States) (Gowda et al., 2017). The sections were examined under a light microscope (E400, Nikon, Tokyo, Japan). To evaluate birefringence pattern of collagen fibers, the sections were examined under 100× magnification using a polarized light microscope.

### Cytokine Quantification

The concentration of cytokines IL-1β, IL-10, and TNF-α was measured using commercial enzyme immunoassay kits (R&D Systems, Minneapolis, Minnesota, United States), and the optical density was measured at 490 nm in a spectrophotometer. The samples were homogenized and processed following an established method (Safieh-Garabedian et al., 1995). Ninety six-well plates were incubated for 12 h at 4°C with the capture of IL-1 β, IL-10, and TNF-α. After sensitization of the plates, the samples were added and incubated for 2 h. The plates were washed three times with wash solution and incubated with biotinylated monoclonal antibody. After the incubation period the plates were washed, and then diluted streptavidin solution was added. The enzymatic reaction was stopped with H_2_SO_4_ (1 M). The results were expressed in pg/mL.

### qPCR

Quantitative RT-PCR analysis was performed using SYBR Green (Applied Biosystems, United States) and Step One Plus (Applied Biosystems, United States) according to the manufacturer’s instructions. Total sample RNA (*n* = 4) was extracted with the Trizol reagent (Life Technologies, CA, United States) as described in the literature (Chomczynski and Sacchi, 2006). Subsequently, the samples were isolated and purified using the total RNA isolation system of SV (Promega Corporation, United States). The RNA concentration was determined by measuring the optical density at a wavelength of 260 nm (OD260) with a density equivalent to 40 μg/ml of RNA. Five milligrams of RNA were reverse transcribed to cDNA using a reaction mixture containing 4 μl of 5x reaction buffer, 2 μl of dNTP mixture (10 mM), 20 units of RNase inhibitor, 200 units of reverse transcriptase and 0.5 μg of the first oligo DT (High-capacity cDNA reverse transcription kit, Foster City, United States) in a total volume of 20 μl. The reaction was subjected to 42°C for 60 min and terminated by heating at 70°C for 10 min.

Expression of the gene was assessed by PCR amplification using primer pairs from *Rattus norvegicus* (GADPH: Forward: AACTTGGCATCGTGGAAGG, Reverse: GTGGATGCAGGGATGATGTTC; VEGF: Forward: GGTTTGGAGAGGTTGCTCCTT; Reverse: CTTTCCTCCTCTGCTGATTTCCAAAA; TGFβ: Forward: GAGGTGACCTGGGCACCAT, Reverse: GGCCATGAGGAGCAGGAA).

### Statistical Analysis

The data are presented as the means ± standard error of the mean. Comparisons between the groups were performed by a unidirectional analysis of variance (ANOVA), and Tukey’s post-test was used to compare the means. GraphPad Prism 5.0 (La Jolla, CA, United States) was used. When the value of *p* ≤ 0.05 was obtained, the differences were considered statistically significant.

## Results

### Synthesis and Membrane Preparation

The[Zn(phen)(van)_2_] (ZPV) complex was obtained by substitution of the chloride ligands in the precursor by two bidentate vanillate ligands originating from an octahedral complex where the methoxy groups of the vanillate ions are disposed opposite to each other in a structural arrangement identified previously as *cis-III* (de Medeiros et al., 2018).

The preparation of chitosan (CS) membranes modified with [Zn(phen)(van)_2_] complex, identified in this work as CS-ZPV, was performed by a condensation reaction between the aldehyde groups of the vanillin ligand and the amine groups distributed in the chitosan structure originating from a Schiff base. Therefore, the complex is chemically bound to the chitosan membrane by the resulting imine group (-HC = N-).

**FIGURE 1:**
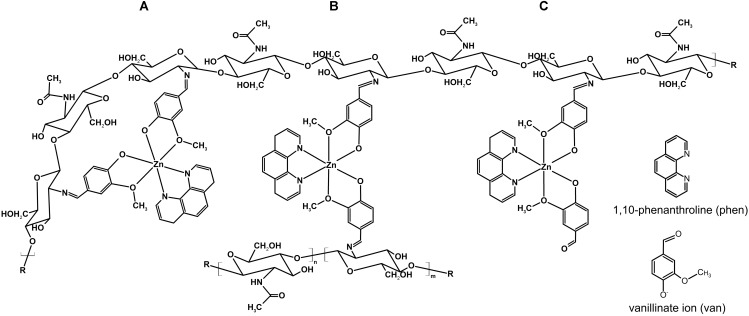
Possible structures resulting from the chitosan modification with [Zn(phen)(van)_2_] (CS-ZPV), **(A)** originating from an intra chain reaction involving both aldehydes present in the ZPV complex, **(B)** resulting from an interchain crosslinking reaction, and **(C)** originating by the reaction of only one of the aldehyde groups available in the complex.

Due the presence of several free amino groups in the polymer, the addition of the [Zn(phen)(van)_2_] to the chitosan structure can occur in three different ways along the polymer chain. Figure 1 shows the structural possibilities in the CS-ZPV membranes. In the first condition, Figure 1A, the two aldehydes of the [Zn(phen)(van)_2_] complex react with amine groups from the D-glucosamine monomer of the same chitosan chain. In the second possibility, Figure 1B, the aldehydes of the complex react with the D-glucosamine monomers of distinct polymer chains in an interchain crosslinking reaction. Finally, there is a third possibility of modification, Figure 1C, in which only one of the aldehydes of [Zn(phen)(van)_2_] react with the primary amine of the D-glucosamine monomer. In this case, there is still a free aldehyde in the molecule.

### Spectroscopic and Electrochemical Characterization of the [Zn(phen)(van)_2_]

The complex [Zn(phen)(van)_2_] was characterized by spectroscopic (infrared, UV-Vis, RMN H^1^) and electrochemical (cyclic and differential pulse voltammetry) techniques.

The infrared spectrum of the complex presented vibrational modes typical of the phenanthroline (phen) ligand as the aromatic C = C stretching (ν), 1586 and 1552 cm^-1^, νC = N at 1426 cm^-1^ and the out-of-the-plane deformations (δ) C-H at 849 and 727 cm^-1^. Additionally, peaks referring to the vanillate ligand were also observed as the intense band referring to νC = O of the aldehyde group at 1660 cm^-1^ and a set assigned to the ether group (1463, 1426, and 1021 cm^-1^).

The electronic spectrum (UV-Vis) of ZPV in aqueous solution presented many absorptions in the ultraviolet region. Four of them, 201, 228, 271, and 292 nm, refer to the π-π^∗^ phenintra ligand transitions as well as the bands at 312 and 345 nm that are assigned to the intra ligand π-π^∗^ vanillate ion. As zinc(II) has a d^10^ configuration, any d-d type transition was observed.

The H^1^ NMR spectrum in DMSO-d6, as shown in has peaks referring to the vanillate ligand as the intense peak at 3.71 ppm referring to the protons of the methoxide group, H_1V_, the doublet at 6.67 ppm of the proton H_2v_ coupled with H_4v_, which has signals at 7.28 and 7.31 ppm. There are also two singlets at 7.24 and 9.57 ppm referring to the protons identified as H_3V_ and H_6V_, respectively. The latter proton presents a greater chemical shift due to the effect of deprotection induced by the carbonyl of the aldehyde. Additionally, the signals at 8.09 (singlet), 8.29 (triplet) and 8.91–8.99 ppm (doublet) refer to the protons of phenanthroline H_d_, H_b_ and H_c_, respectively.

The cyclic voltammogram of [Zn(phen)(van)_2_], in 0.1 mol L^-1^ PBS, pH 7.4, showed one irreversible anodic process at 657 mV (vs. Ag/AgCl) referring to the oxidation of the vanillate ion coordinated with the metal. This value is slightly lower than the oxidation potential of the free vanillin in the same experimental conditions at 675 mV (vs. Ag/AgCl).

These results are in agreement with the data obtained for the similar copper system, [Cu (phen)(van)_2_], present in the literature (de Medeiros et al., 2018).

### Spectroscopic and Electrochemical Characterization of the Modified Chitosan Membrane

The infrared spectrum of the purified chitosan membrane shows typical bands for this polymer as the symmetric and asymmetric C-H stretching (ν) at 2920 and 2879 cm^-1^, νC = O at 1652 cm^-1^, and νNH of the amines at 1586 cm^-1^. There are also bands at 1420 and 1378 cm^-1^, attributed to the C-H angular deformation, and others assigned to νCH_3_ of the amide, at 1321 and 898 cm^-1^ (Stoica et al., 2010).

**FIGURE 2:**
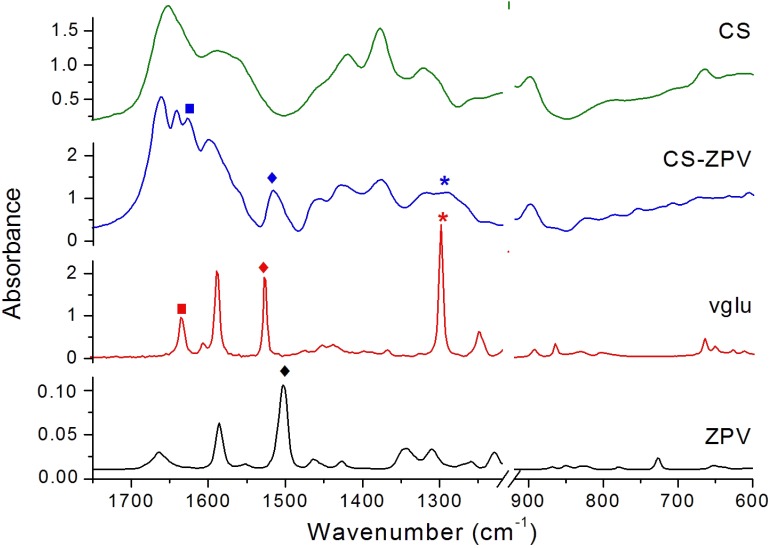
Infrared spectra of [Zn(phen)(van)_2_] (ZPV) complex (black), the Schiff base vglu (red), the chitosan membrane modified with [Zn(phen)(van)_2_], CS-ZPV (blue), and chitosan (CS) membrane (green). Symbols represent the ν_C__=__N_(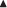), ν_C_ = _C_ of phenanthroline and vanilloid group (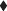) and δ_C-H_ and δ_O-H_ of the glucosidic ring (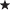). The region of the glycosidic ring νC-O-C and β-(1-4) glycosidic bridge, from 1218 to 919 cm^-1^, were omitted to allow a better view of less intense bands.

The infrared spectra of the modified chitosan membranes, CS-ZPV1 and CS-ZPV2, also presented the same vibrational modes related to the polymeric matrix. However, five new bands at 1662, 1627, 1516, 1292, and 754 cm^-1^ were also observed, as shown in Figure 2. The absorption at 1662 cm^-1^ refers to the aldehyde group (νC = O) of the vanillate ion. The presence of this band in the IR spectrum indicates the presence of free aldehydes in the membrane as proposed in the structure of Figure 1C.

The peak at 1627 cm^-1^, typical of the νC = N of the imine, confirms the chemical modification of the chitosan membrane and synthesis of the new Schiff base CS-ZPV. Additionally, the νC = C of the aromatic rings (phenanthroline and vanillate ion) was observed at 1516 cm^-1^, while the band at 1292 cm^-1^ was assigned to the C-H and O-H deformation of the glucosamine ring. The absorption at 754 cm^-1^ refers to the out-of-plane δC-H of the phenanthroline rings present in the ZPV structure.

To better understand the structure of the modified chitosan, we also investigated a simplified structural portion of the CS-ZPV membrane obtained through the synthesis of the Schiff base from the reaction between vanillin and D-glucosamine, resulting in the compound 2-deoxy-2-[(E)-(4-hydroxy-3-methoxybenzylidene)amino]hexopyranose, identified here as vglu. A comparative of the infrared spectra of CS-ZPV and vglu can be found in Figure 2.

Clearly, only the CS-ZPV membranes have the νC = O of aromatic aldehyde from the vanillin, 1662 cm^-1^. The νC = N at 1626 cm^-1^, present in the vglu spectrum, was also observed in the CS-ZPV2 without significant shift. It is further verified that the bands at 1516 and 1292 cm^-1^ on the zinc membrane were also present in the vglu spectrum (1526 and 1298 cm^-1^) with small shifts due the coordination with the metal.

### Scanning Electron Microscopy

**FIGURE 3:**
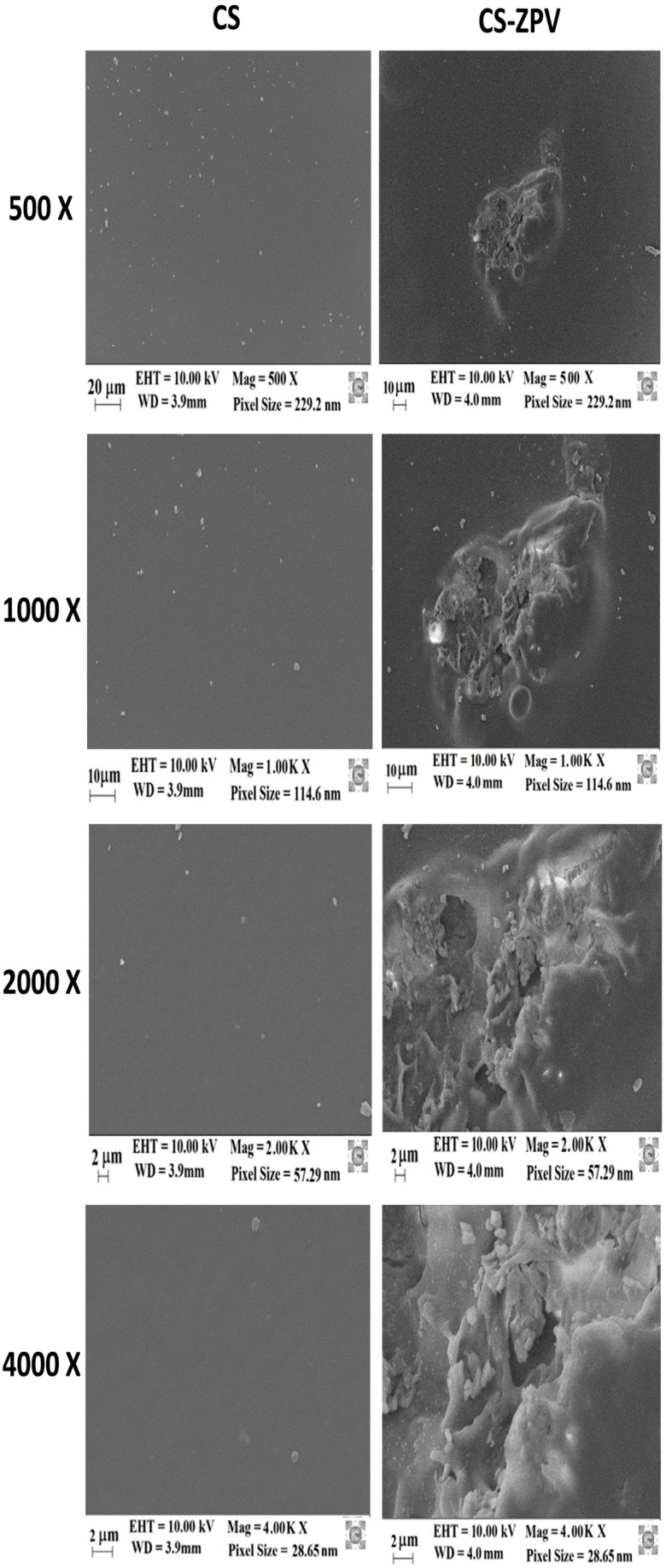
Scanning electron microscope images of CS and CS-ZPV membranes. Magnifications of 500, 1000, 2000, and 4000×.

The imine bond formation on the chitosan structure implies a modification of the membrane surface morphology. Thus, we evaluated these modifications comparing the scanning electron microscopy (SEM) images of the pure (CS) and modified chitosan membranes (CS-ZPV). Figure 3 shows the SEM images with magnifications of 500, 1000, 2000, and 4000×.

The CS membrane has a homogeneous, smooth and continuous structure with the absence of macropores. These results characterize the membrane as dense, and they are consistent with the data obtained by other authors (Zhang et al., 2015). Only a few particles, possibly related to the sodium hydroxide from the washing process, remaining on the surface of the membrane are perceptible.

The morphological analysis of the CS-ZPV2 membranes shows a smooth surface, with agglomerations of dispersed particles with regular spacing. Some of these agglomerates intercalate the surface of the membrane and, therefore, are partially covered. When observing the magnification of 4000×, it is verified that the particles present fragile characteristics, with a fragmented aspect, as observed in Figure 3 for the CS-ZPV2 membrane. These agglomerated particles are not present in the pure chitosan membrane and therefore are indicative of the modification of the polymeric membrane.

An Energy Dispersion System (EDS) coupled to the SEM was also used to qualitatively determine the composition of the metals present in the modified chitosan membrane (CS-ZPV2). The EDS graph of CS-ZPV2 presented in obtained for the membrane region presented peaks related to oxygen (0.5 KeV), zinc (close to 1 and 9 KeV) and gold (at 2 KeV). Of these elements, oxygen and zinc were present in the structure of the ZPV complex bound to the polymer chain, indicating the modification of the membrane, whereas the gold comes from the metallization of the membrane.

### Evaluation of Delivery and Quantification of the [Zn(phen)(van)_2_] Content in the Modified Chitosan Membrane

UV-Vis spectroscopy was used to monitor the hydrolysis reaction of the modified chitosan and consequent release of the [Zn(phen)(van)_2_] from the membrane. The technique was used also to determine the concentration of the zinc complex present in the membrane.

A square piece of the CS-ZPV2 membrane with an area of 0.08 cm^2^ was immersed in 3.2 mL of a PBS solution, 0.1 mol L^-1^, pH 7.4, at 25°C. Several electronic spectra of the solution were recorded at different immersion times in the interval of 0–1305 min.

**FIGURE 4:**
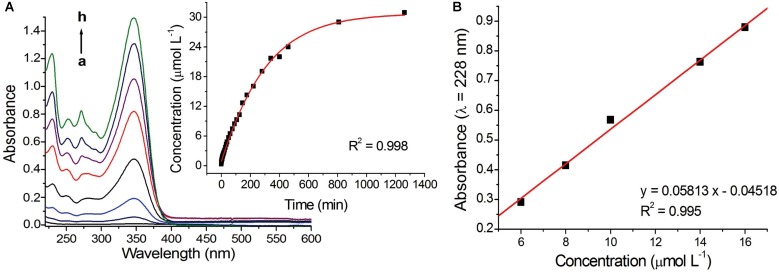
**(A)** Representative spectra of the PBS solution, 0.1 mol L^-1^, pH 7.4 at 25°C, obtained after different times of immersion of the modified membrane CS-ZPV2 (t_a_ = 0 min, t_b_ = 1 min, t_c_ = 5 min, t_d_ = 15 min, t_e_ = 30 min, t_f_ = 60 min, t_g_ = 120 min, t_h_ = 1305 min). The inset present the kinetic curve for all obtained data. **(B)** Calibration curve of [Zn(phen)(van)_2_].

Interestingly, there was a gradual increase in the absorbance of the bands typical of the ZPV complexes with the time of exposure of the membrane to the aqueous solution, as shown in Figure 4A. This result clearly indicates that the method applied to modify the chitosan membrane was effective and confirms the capacity of the membrane to deliver the zinc complex when exposed to water. After 809 min of monitoring, the concentration of the complex remained practically constant in the solution. A calibration curve (Figure 4B) built for the [Zn(phen)(van)_2_] complex allowed the concentration of the compound released into the solution by the membrane at each time evaluated to be determined, and it is presented in the kinetic curve of the inset in Figure 4A, resulting in a concentration of 3.06 × 10^-5^ mol L^-1^ at *t* = 1064 min.

Considering the superficial area of the membrane evaluated and the concentration of the zinc(II) complex released in the UV-Vis experiment, the total concentration of complex present in the CS-ZPV1 membrane was 0.736 μmol cm^-2^ (or 0.403 mg cm^-2^) while CS-ZPV2 resulted in a concentration per area of membrane of 1.22 μmol cm^-2^ (or 0.671 mg cm^-2^). The capacity of the modified membrane to release the zinc complex in aqueous solution was also monitored by electrochemical techniques. The cyclic and differential pulse voltammograms obtained after different immersion times of the CS-ZPV films in 0.1 mol L^-1^ PBS, pH 7.4, show only one oxidation process referring to the coordinated vanillin ligand (620 mV vs. Ag/AgCl). Similarly, to the trend observed in the monitoring performed by UV-Vis, the anodic peak current also increased with the immersion time, indicating the release of the modified membrane complex.

### Macroscopic and Histopathological Analysis

**FIGURE 5:**
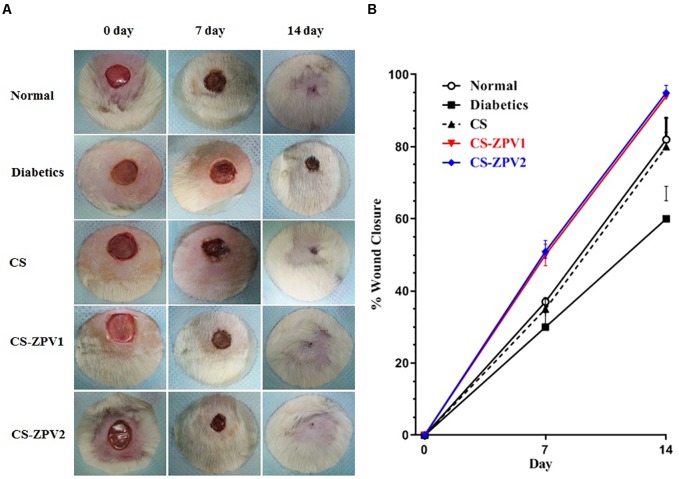
**(A)** Effects of CS-ZPV on wound healing in diabetic rats. Macroscopic analysis of skin ulcers on days 0, 7, and 14 after treatment with the chitosan membrane (CS), zinc-vanillin complex membrane CS-ZPV1 (0.403 mg cm^-2^) or CS-ZPV2 (0.671 mg cm^-2^). **(B)** Wound closure is expressed as a percentage closure relative to original size on the 7th and 14th postoperative day according to the treatment received.

Macroscopic evaluation of skin lesions (Figure 5) on the 7th and 14th postoperative day showed a significant difference regarding the area of the lesions, which was approximately 35 and 66% for the normal group (without diabetes, with natural cure), 20 and 62% for diabetic animals without treatment, 40 and 70% for CS (diabetic, treated with isolated chitosan membrane), 51 and 98% in the CS-ZPV1 treatment and 63 and 99% in the CS-ZPV2 treatment on the 7th and 14th days, respectively. In the comparison between means, the groups treated with the chitosan membrane and zinc complex at doses 01 and 02 did not show any significant difference between them, but they were statistically superior to the other groups.

**FIGURE 6:**
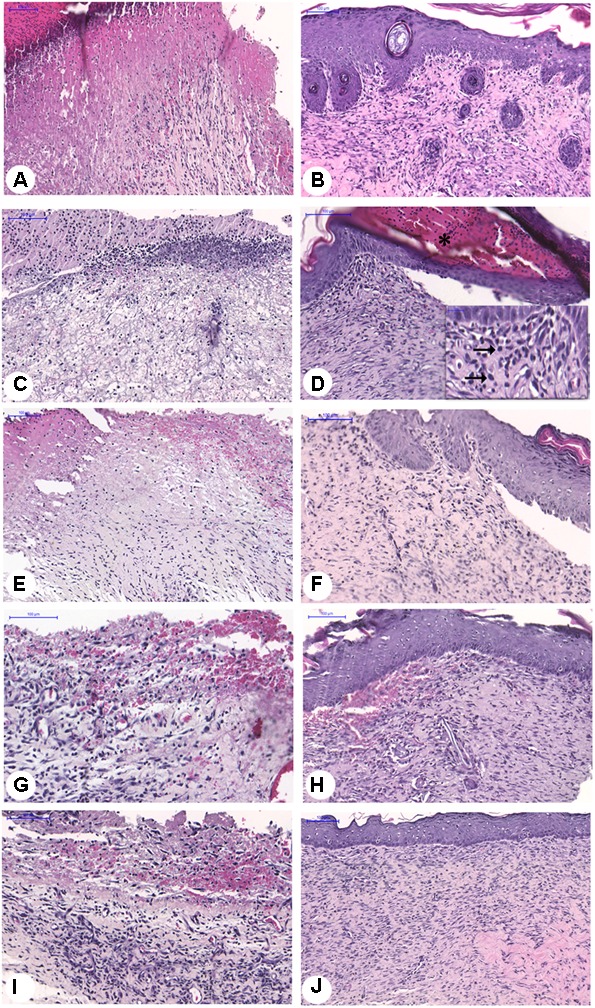
Histopathological analysis of ulcer of diabetic rats at 7 or 14 experimental days, Normal/Natural cure – 7 days **(A)** or 14 days **(B)**. Diabetic/untreated rats – 7 days **(C)** or 14 days **(D)**, diabetic rats treated with CS-7 days **(E)** or 14 days **(F)** membrane. Diabetic rats treated with CS-ZPV membrane – 7 days **(G)** or 14 days **(H)**; Diabetic rats treated with CS-CZPV2 membrane – 7 days **(I)** or 14 days **(J)**. Black horizontal arrows point to mononuclear cells.

In the histopathological analysis HE (Figure 6), the remodeling of the dermis and the closure of the wound were observed to be complete in the normal animals. The wounds from untreated diabetic animals had incomplete reepithelialization. The animals treated with the zinc-vanillin complex showed improvement of ulcer healing, as evidenced by the presence of densely collagenated fibrous connective tissue, and discrete inflammatory infiltrate compared to that of the untreated diabetic animals.

**FIGURE 7:**
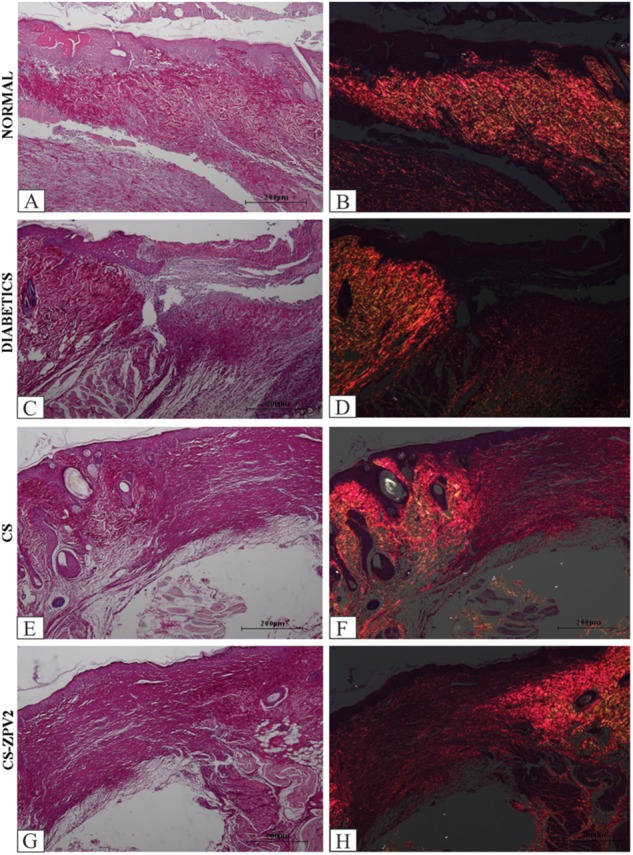
Histopathological analysis of the lesion of diabetic rats after staining with Picrosirius and under polarized light on the 14th day of the experimental model. Normal group, without diabetes **(A,B)**, diabetic without treatment **(C,D)**, rats with diabetes treated with chitosan (CS) **(E,F)** and diabetic animals treated with a zinc-vanillin complex membrane dose of 0.671 mg cm^-2^ (CS-ZPV 2) **(G,H)**. The presence of type I collagen fibers was observed. Increase (×200).

**FIGURE 8:**
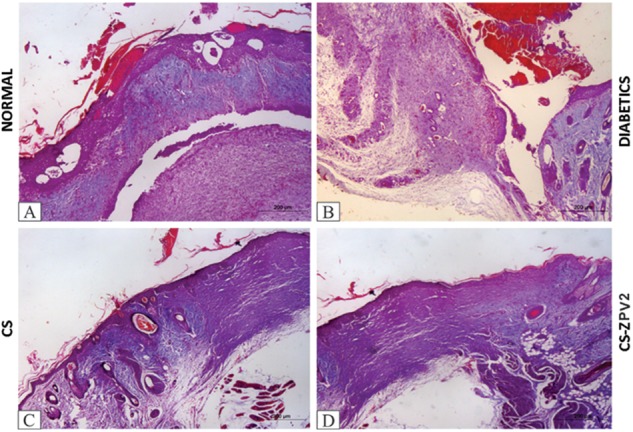
Histopathological analysis of the lesion of diabetic rats after staining with Mallory’s trichrome on the 14th day of the experimental model. Normal group without diabetes **(A)**, diabetic animals without treatment **(B)**, diabetic animals treated with chitosan (CS), **(C)** and diabetic animals treated with a zinc-vanillin complex membrane dose of 0.671 mg cm^-2^ (CS-ZPV 2) **(D)**. Blood vessels stained in red were evidenced. Increase (×200).

In the rats treated with the CS-ZPV membrane, collagen fibers were similar to the normal group, as observed in HE and picrosirius red-stained slides, and under polarized light the collagen fibers showed an increase in reddish-yellow birefringence, characteristic of type I collagen (Figure 7). In the staining with Mallory’s trichrome, blood vessels were observed in the groups treated with the CS-ZPV membrane complex (Figure 8).

### IL-1β, TNF-α, and IL-10 Cytokine Assays

**FIGURE 9:**
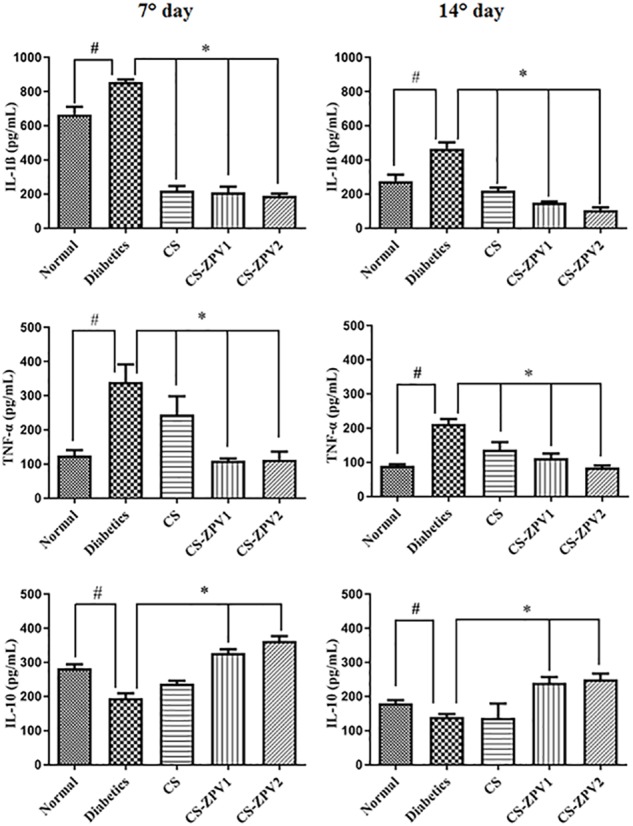
Levels of interleukin IL-1β, tumor necrosis factor (TNF-α) and interleukin IL-10 on day 7 or 14 of the experimental model of healing in diabetic rats. The normal group comprised animals without diabetes and treatment. The diabetic group comprised rats that were not treated. The diabetic group comprised animals treated with membrane chitosan pure (CS). The diabetic group comprised rats treated with membrane chitosan with complex zinc-vanillin (CS-ZPV1 and CS-ZPV2) at two different doses (0.403 mg cm^-2^ or 0.671 mg cm^-2^). The results are presented as the mean ± standard error of the mean (*n* = 5). ^∗^*p* < 0.05 vs. the group normal, ^#^*p* < 0.05 vs. the diabetics group (analysis of variance with Tukey’s post-test).

The untreated diabetic group had elevated IL-1β and TNF-α cytokine levels at 7 and 14 days compared to those of the normal group (Figure 9). The CS-ZPV-treated groups at doses 1 and 2 had lower levels of IL-1β and TNF-α cytokines at 7 and 14 days than those the untreated diabetes group. The untreated diabetic group exhibited lower IL-10 levels than that of the normal group (*p* < 0.05). The groups treated with chitosan membrane and zinc-vanillin complex reported significant (*p* < 0.05) IL-10 increases levels compared to those of the untreated diabetic group (Figure 9).

### RT-PCR for TGF-β and VEGF

**FIGURE 10:**
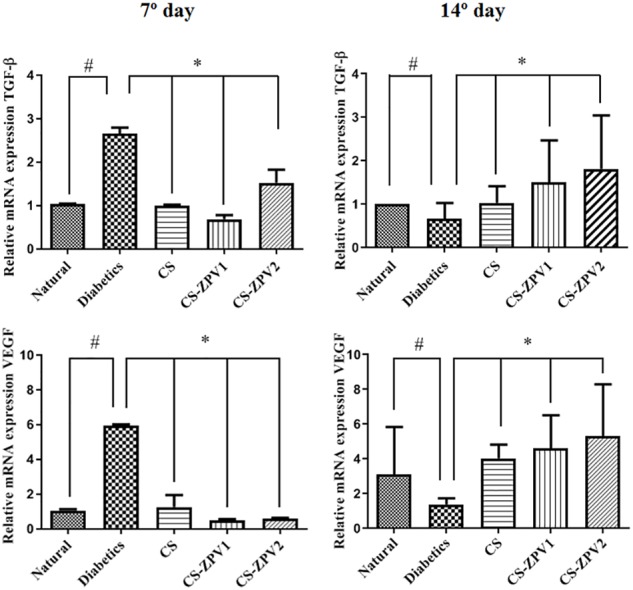
Real-time polymerase chain reaction for transforming growth factor beta (TGF-β) and vascular endothelial growth factor (VEGF) on day 7 or 14 of the experiment. The diabetic group treated with CS-ZPV dose 2 exhibited decreased gene expression for TGF-β and VEGF compared with the normal group (*n* = 5; ^∗^*p* < 0.05 vs. the normal group, ^#^*p* < 0.05 vs. group diabetic, untreated; analysis of variance with Tukey’s post-test).

The diabetic group without treatment had lower mRNA expression of TGF-β than the normal group on the 14th day of the experiment model. For VEGF, the untreated diabetic group exhibited an increase in mRNA expression compared with the normal group (Figure 10). The diabetic group treated with CS-ZPV 2 showed increased mRNA expression of TGF-β and VEGF on the 14th day of the experiment (Figure 10, *p* < 0.05). On the 7th day of the experimental model, significant differences were not observed.

## Discussion

The new octahedral zinc(II) complex, [Zn(phen)(van)_2_], with the bidentate ligands 1,10-phenanthroline (phen) and vanillate ions (van), was synthesized and characterized by spectroscopic and electrochemical techniques. The results corroborate the proposed structure and are consistent with the ones previously reported for a copper(II) complex having the same set of ligands (de Medeiros et al., 2018).

The *cis*-like arrange of the two vanillate ions around the metal in the ZPV structure make the aldehyde groups available for additional reactions. Particularly, the reactivity of aldehyde with primary amines, originating from the Schiff bases, was explored in this work to chemically modify the chitosan structure.

The traditional procedure of chitosan derivatization is based on a homogeneous condition where an aqueous solution of a compound is added to a chitosan solution in 1% acetic acid and stirred for a long period, followed by evaporation of the solvent and subsequent washing of the film with sodium hydroxide solution. Differently, we developed a heterogeneous methodology, initially preparing the chitosan films and then subjecting the membrane to a reaction with the compound solubilized in an organic solvent. This procedure avoids exposing the complex to the extreme pH conditions used in the membrane preparations, which could lead to degradation of the compound. In addition, this method prevents the hydrolysis of the imine because it is performed in the absence of water.

Thus, the chitosan membrane was immersed in ZPV solutions for 14 and 20 days to obtain the membranes identified as CS-ZPV1 and CS-ZPV2. The infrared spectra of the membranes presented bands referring to the imine bond, νC = N, at 1627 cm^-1^ and to the phenanthroline ringat 754 cm^-1^. In addition, the SEM images showed that the surface morphology was drastically altered after reaction with the complex, as evidenced by the increase in roughness of certain areas of the membrane. The EDS analyses confirmed the presence of zinc in these areas. These results confirmed the chemical modification of the chitosan by introduction of ZPV complex in a Schiff base reaction.

The Schiff bases, in general, present low stability when in aqueous medium. The hydrolysis process of these compounds originates again from their precursor molecules (Abramov et al., 2007). This chemical characteristic has been explored for the development of drug delivery systems under specific conditions of the organism (Cheng et al., 2008). In this sense, we monitored the release of [Zn(phen)(van)_2_] compound by UV-Vis spectroscopy. The results showed that the modified membranes release the zinc compound gradually when subjected to an aqueous medium (PBS solution). The complete release of complex was achieved after 932 min of immersion of the CS-ZPV1 and 1064 min for the CS-ZPV2.

From the kinetic plot and the calibration curve it was possible to determine the concentration of complex in the membranes, which was 0.403 mg cm^-2^ for CS-ZPV1 and 0.671 mg cm^-2^ for CS-ZPV2.

In the present study, the chitosan membranes modified with [Zn(phen)(van)2] complex improved wound healing in diabetic rats, as evidenced by the reduction in the area of the lesion and the histopathological findings. The animals treated with the CS-ZPV complex showed an improvement in ulcer healing, as evidenced by the presence of type I collagen fibers, areas of reepithelialization, blood vessels and discrete inflammatory infiltrate. Zinc compounds have been recognized as an important metal in the wound healing process (Andrews and Gallagher-Allred, 1999; Lin et al., 2017). Chitosan has also been successfully applied for the same proposal with relevant results (Martínez-Camacho et al., 2010; Zou et al., 2015) in addition to its wide use as a matrix of drug delivery systems (Martínez-Camacho et al., 2010). In this work, we combined the properties of these substances through the development of a new chitosan membrane modified with [Zn(phen)(van)_2_] complex and evaluated the delivery capacity of compound in an aqueous medium and the wound healing activity in diabetic rats. Other authors corroborate the data obtained in this research, where dressings or compounds based on zinc and or chitosan increased wound healing in rat models (Kumar et al., 2012; Cassettari et al., 2013, 2014).

Several processes are involved in wound healing, including inflammation, coagulation, reepithelialization and neovascularization (Pakyari et al., 2013). In the inflammatory phase, neutrophils are the first cells to migrate to the wound site, and pro-inflammatory cytokines IL1-β and TNFα participate in the process (Behm et al., 2012). In the present study, the untreated diabetic group showed an intense inflammatory infiltrate and elevated levels of pro-inflammatory cytokines (TNF-α, IL-1β). The animals treated with the CS-ZPV complex reversed these parameters. TNF-α inhibits the healing of cutaneous wounds, which may interfere with the expression of type I collagen and reduce the action of fibroblasts induced by transforming growth factor beta (TGF-β) (Goldberg et al., 2007; Lai et al., 2009). Modulation of IL1-β accelerates the wound healing process (Oryan et al., 2018). In accordance, Mallory and picrosirius-stained skin sections of CS-ZPV-treated groups showed a greater collagen deposition, mainly type I, in the dermis of the wound region, compared to the untreated diabetes group. In addition, we have shown that CS-ZPV complex increases IL 10 levels, an established anti-inflammatory cytokine. This result is in accordance with data from the literature, where authors suggest that IL-10 inhibits pro-inflammatory cytokines and increases wound healing (Wang and Wang, 2009).

Other data from this research showed that the animals treated with the chitosan-zinc complex had increased mRNA expression for TGF-β and VEGF on day 14 of the experimental model. TGF-β acts on serine/threonine kinase type I or II receptors expressed on the cell surface (Shi and Massague, 2003). The authors demonstrated the role of TGF-β in wound healing (Pakyari et al., 2013; Denton et al., 2009). At the intermediate stage, TGF-β stimulates the formation of granulation tissue and the expression of extracellular matrix components, mainly fibronectin, collagen types I and III, and VEGF (Barrientos et al., 2008). However, studies of the effects on the expression of these proteins are necessary to confirm the participation of TGF-β and VEGF associated with the protective effects of CS-ZPV complex in the model of skin wound healing in diabetic rats. VEGF represents a marker of healing cutaneous wounds as a proangiogenic molecule of the skin. In endothelial cells, VEGF signals angiogenesis and may act on the keratinocytes stimulating reepithelialization and assisting in the resolution of inflammation (Johnson and Wilgus, 2014).

**FIGURE 11:**
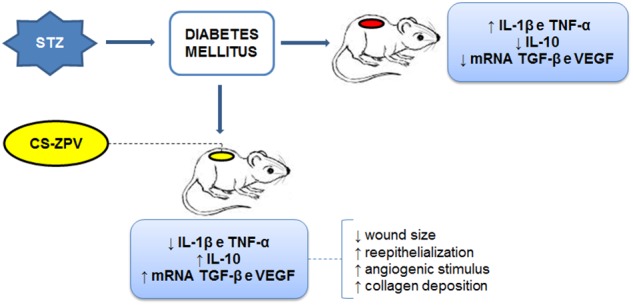
Synthesis of the results obtained in the pharmacological modulation. Streptozotocin (STZ) was able to induce diabetes in the animals, and then the lesion was performed on the back of *Wistar* rats. The levels of IL-1β, TNF-α, IL-10, and gene expression of TGF-β and VEGF were observed. The CS-ZPV membrane (the chitosan membrane modified with [Zn(phen)(van)2] complex) contributed to the decrease in pro-inflammatory factors and better wound healing.

In conclusion, the CS-ZPV complex improved healing in diabetic rats by reducing the levels of the pro-inflammatory cytokines TNF-α and IL-1β, increasing the IL-10 cytokine content and promoting collagen formation, reepithelialization and neovascularization. Increased gene expression of TGF-β and VEGF was observed (Figure 11). However, more studies are necessary.

## Author Contributions

EA, WM, JG, RC, CS, and CA: experimental design. EA, WM, AA, RC, RF, and CA: investigation. EA, WM, TP, MM, AA, RF, JG, RC, CS, FO, AC, DL, and CA: methodology. EA, WM, RC, DL, CA, and MM: writing of the manuscript. AA, RC, RF, DP, CA, and AC: revision of the manuscript.

## Conflict of Interest Statement

The authors declare that the research was conducted in the absence of any commercial or financial relationships that could be construed as a potential conflict of interest.
